# Impact of tailored diabetes education on adherence and glycemic control in children and adolescents on continuous subcutaneous insulin infusion, prospective interventional study in a tertiary center

**DOI:** 10.1186/s12887-025-06018-4

**Published:** 2025-09-01

**Authors:** Mona Hassan, Mahmoud ElHelaly, Nora Badawi, Mariam Nader, Radwa Shamma

**Affiliations:** 1 Department of Pediatrics, Faculty of Medicine, Cairo University, Cairo, Egypt; 2 Department of Pediatrics, Military Medical Academy, Cairo, Egypt; 3 Department of Pediatrics, Faculty of Medicine, New Giza University, Giza, Egypt

**Keywords:** Type 1 diabetes, Insulin pump, Continuous subcutaneous insulin infusion, Diabetes education, Glycemic control, Pediatric diabetes

## Abstract

**Background:**

Structured diabetes education plays a crucial role in the management of Type 1 Diabetes (T1D), especially with advanced technologies such as continuous subcutaneous insulin infusion (CSII). This study evaluates the impact of tailored education on adherence and glycemic control among pediatric CSII users.

**Patients and methods:**

This interventional analytical study included 30 patients diagnosed with T1D with age ranged from 5 to 18 years, recruited from Diabetes & Endocrinology Clinic at tertiary university-based children’s hospital. Patients were recruited for one year duration. Patients who were on multiple daily injection (MDI) for 2 to 10 years were shifted to CSII after receiving comprehensive diabetes education. Their education level was assessed individually using a questionnaire to assess patients’ knowledge and application skills at the time of insulin pump insertion, 3 and 6 months later. Twenty-five patients were on mini-med 715, 10 patients used flash glucose monitoring, and 15 patients used self-monitoring blood sugar, while five patients were on mini-med 780G and continuous glucose monitoring (CGM) using guardian 4 (G4).

**Results:**

Study included 19 females (63.3%) and 11 males (36.7%) with age ranged from 7 to 11 with mean ± SD of 10.74 ± 1.86. Patients’ education level was assessed before pump insertion, and 3 months later which shows significant improvement in patients’ diabetes knowledge and technical skills with P-value 0.038. Also, time in range (TIR) improved significantly in well-educated patients at 3 and 6 months with P-values 0.048 and 0.025 respectively. There was highly significant improvement of HbA1c after 6 months (8.82 ± 1.5 vs. 7.18 ± 0.56) with P-value 0.000 after receiving diabetes education.

**Conclusions:**

Tailored diabetes education significantly improves adherence and glycemic outcomes in pediatric patients using CSII. Integrating structured educational programs with diabetes technology is essential to achieve optimal metabolic control in youth.

## Background

Type 1 diabetes (T1D) is a complex, chronic autoimmune disease that typically manifests in childhood and adolescence, requiring lifelong insulin therapy and comprehensive care. Managing T1D involves not only maintaining glycemic control but also addressing psychosocial factors, preventing acute and chronic complications, and integrating evolving technologies into daily life [1].

The pace of development in diabetes technology is extremely rapid. Having a device or application does not change outcomes unless human being engages with it to create positive health benefits. This emphasizes the need for the health care team to assist people with diabetes in device and program selection and to support their use through ongoing education and training [2].

Continuous Subcutaneous Insulin Infusion (CSII), also known as insulin pump therapy, has emerged as a key advancement in diabetes care, offering improved glycemic variability, lifestyle flexibility, and reduced hypoglycemic episodes. However, the benefits of CSII are closely linked to the patient’s ability to manage the device effectively, which hinges on comprehensive and tailored diabetes education.

Educational interventions are essential to enhance patient and caregiver knowledge, develop necessary technical skills, and foster adherence to insulin regimens. The International Society for Pediatric and Adolescent Diabetes (ISPAD) and the American Diabetes Association (ADA) emphasize the importance of structured education programs in achieving optimal clinical outcomes with technologies like CSII and Continuous Glucose Monitoring (CGM).

Despite global evidence on the benefits of education, there is limited data from our country on how tailored diabetes education impacts adherence and glycemic control in pediatric patients using insulin pumps. This study aims to bridge that gap by evaluating the effect of individualized diabetes education on metabolic outcomes and behavioral adherence among children and adolescents transitioned from Multiple Daily Injections (MDI) to CSII therapy.

## Patients and methods

### Study design and setting

This was a prospective interventional analytical study conducted over one year duration at the Diabetes & Endocrinology Clinic at tertiary university-based children’s hospital.

### Participants

The study enrolled 30 children and adolescents (aged 5–18 years) diagnosed with T1D who had been on MDI therapy for 2 to 10 years. Then shifted to CSII after receiving comprehensive diabetes education sessions. Education sessions were delivered by qualified dietitians, and diabetes education nurses, under supervision of clinical diabetologists and endocrinologists.

### Intervention: tailored diabetes education

Upon CSII initiation, patients and caregivers underwent individualized diabetes education sessions. The initial education phase commonly included 3–5 sessions over the course of the first 1–2 weeks, each lasting 30–60 min, depending on the patient’s age, cognitive development, and emotional state. Following this, reinforcement sessions were provided at regular intervals—usually every 3 to 6 months during routine clinic visits—or more frequently if needed. Additional sessions were often scheduled around transitions such as school entry, puberty, or initiation of new treatment modalities (e.g. insulin pumps or continuous glucose monitors).

Age-appropriate, culturally suitable educational materials were used, including illustrated booklets, videos, mobile applications, and hands-on demonstrations. Younger children benefit from visual aids and storytelling, while adolescents may engage more with peer discussions and real-life scenario simulations.

The educational content focused on:

- Blood glucose monitoring.
- Hypoglycemia and hyperglycemia management.
- Carbohydrate counting, insulin dosing including Insulin carbohydrate ratio (ICR) and Insulin sensitivity factor (ISF).
- Skin care and infusion site management.
- Importance of adherence and self-monitoring.
- Sick day management.
- Physical activity considerations.

Device training is a critical component of pediatric diabetes education. Hands-on demonstrations of blood glucose meters, insulin pumps, and continuous glucose monitoring (CGM) systems were conducted. Children and caregivers were guided through:

- Set-up and calibration.
- Site rotation techniques.
- Interpretation of glucose trends.
- Troubleshooting alarms and errors.

The education team included certified diabetes educators, pediatric endocrinology nurses, registered dietitians, and clinical psychologists—all of whom had specialized training in pediatric diabetes care under complete supervision of diabetologists. Many educators hold additional credentials such as Certified Insulin Pump Trainer. Education was provided in both individual and group settings, and team members collaborated closely to ensure consistency of information and support.

Education was highly individualized and adjusted based on several factors including the child’s age, developmental stage, psychosocial background, family structure, literacy level, and learning preferences. For example:

- Toddlers and preschoolers require parent-directed education with focus on routines, safety, and caregiver consistency.
- School-aged children can begin to participate in self-management tasks with guided supervision.
- Adolescents often benefit from autonomy-building strategies, motivational interviewing, and transition readiness planning for adult care.

A family-centered approach is essential, with parents and caregivers included in all educational efforts. In some cases, tele-education platforms were utilized to ensure accessibility and continuity.

### Devices used

- Twenty-five patients (83.3%) used MiniMed Paradigm 715 insulin pumps.
- Five patients (16.7%) used MiniMed 780G pumps with Guardian 4 CGM.
- Ten used flash glucose monitoring (FGM).
- Fifteen relied on self-monitoring of blood glucose (SMBG).

### Data collection

Data was collected at three time-points:

- Baseline (before CSII initiation).
- 3 months after CSII and education.
- 6 months after CSII and education.

Parameters assessed:

- HbA1c levels.
- Random Blood Sugar (RBS) pre- and post-meal.
- Time-in-Range (TIR) from CGM data (for eligible users).
- Diabetes knowledge via structured questionnaire, quoted from International Society for Pediatric and Adolescent Diabetes (ISPAD) guidelines [3].

### Statistical analysis

Statistical analysis was performed using Statistical Package for the Social Sciences (SPSS). Quantitative variables were expressed as mean ± standard deviation. Paired t-tests and ANOVA were used to compare variables across time points. A p-value < 0.05 was considered statistically significant.

## Results

The mean age was 10.74 ± 1.86 years. There was a female predominance with 63.3% females (*n* = 19) and 36.7% males (*n* = 11). Demographic characteristics of patients are shown in Table 1.

**Table 1:** Gender, onset of diabetes, age at pump insertion, presentation on first attack, type of basal insulin and type of bolus insulin among the studied patients (No = 30)

		No. = 30
Gender	Female	19 (63.3%)
	Male	11 (36.7%)
Onset of diabetes (Years)	Mean ± SD	8.34 ± 2.57
	Median (IQR)	8.45 (6–10)
	Range	4–14
Age at PUMP Insertion (Years)	Mean ± SD	10.74 ± 1.86
	Median (IQR)	11 (9–12)
	Range	7–14
Presentation on first attack	DKA	22 (73.3%)
	Hyperglycemia	8 (26.7%)
Type of basal insulin	Degludec	13 (43.3%)
	Glargine100	17 (56.7%)
Type of bolus insulin	Aspart	19 (63.3%)
	Lispro	11 (36.7%)

All patients transitioned from MDI to CSII therapy. Twenty-five (83.3%) patients used the MiniMed Paradigm 715, and 5 (16.7%) patients used the MiniMed 780G with Guardian 4 CGM sensors.

The educational level of the patients and their caregivers was evaluated 3 months after receiving diabetes education sessions using structured questionnaires. Twenty-eight were properly educated, while 2 needed revision of their knowledge. There was a statistically significant difference between them with p-value 0.031 as shown in Table 2.

**Table 2:** Comparison between studied patients 3 months after receiving diabetes education (No = 30)

		Education (after 3 months)				P value
		Well educated		Need more education		
		N	%	N	%	
Education (baseline)	Well educated	22	78.6%	0	0.0%	0.031*
	Need more education	6	21.4%	2	100.0%	

P-value > 0.05: Non-significant; P-value < 0.05: Significant; P-value < 0.01: Highly significant

*: McNemar test

Some parameters improved after shifting from MDI to CSII as ICR and ISF with p-values < 0.001 as shown in Table 3.

**Table 3:** Comparison between MDI and CSII regarding ICR and ISF among the studied patients (No = 30)

		MDI	PUMP	Difference	t-Test value	P-value	Sig.
				Mean ± SD			
ICR	Mean ± SD	14.93 ± 2.46	16.70 ± 2.32	1.77 ± 1.67	−5.777•	< 0.001	HS
	Median (IQR)	15 (15–15)	17 (15–18)				
	Range	10–20	12–22				
ISF	Mean ± SD	66.00 ± 21.11	74.67 ± 19.43	8.67 ± 9.73	−4.878•	< 0.001	HS
	Median (IQR)	60 (50–80)	80 (60–80)				
	Range	40–130	50.00–120.00				

P-value > 0.05: Non-significant; P-value < 0.05: Significant; P-value < 0.01: Highly significant

•: Paired t-test

**Fig. 1:**
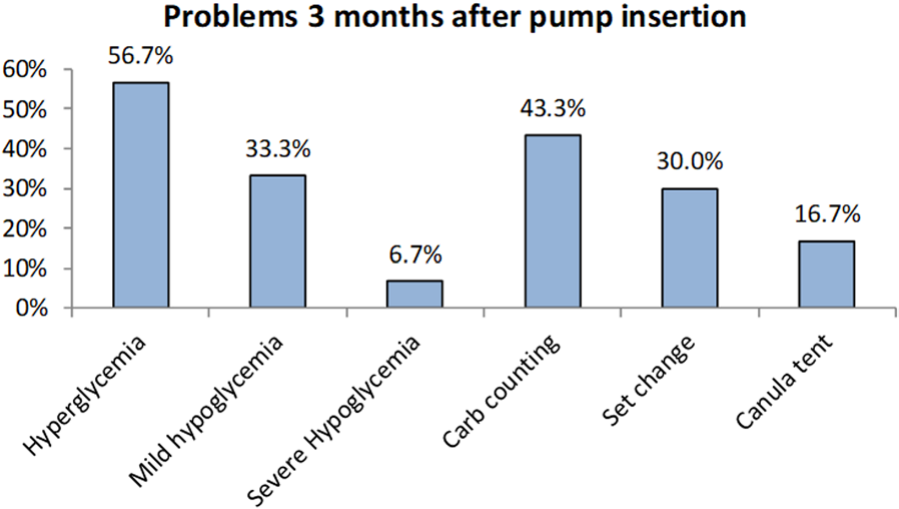
Problems at 3 months after pump insertion among the studied patients

However, there were some problems encountered after pump insertion. Hypoglycemia (33.3% developed mild hypoglycemia with RBS from 54 to 70 mg/dl, 6.7% developed serious hypoglycemia with RBS < 54 mg/dl) and hyperglycemia are relatively common, occurring in 56.7% of studied patients. Issues such as canula tenting and difficulties with carbohydrate counting and set changes also affect a considerable number of studied patients, which were presented by 16.7%, 43.3% and 30.0% of the study participants respectively as shown in Fig. 1.

**Fig. 2:**
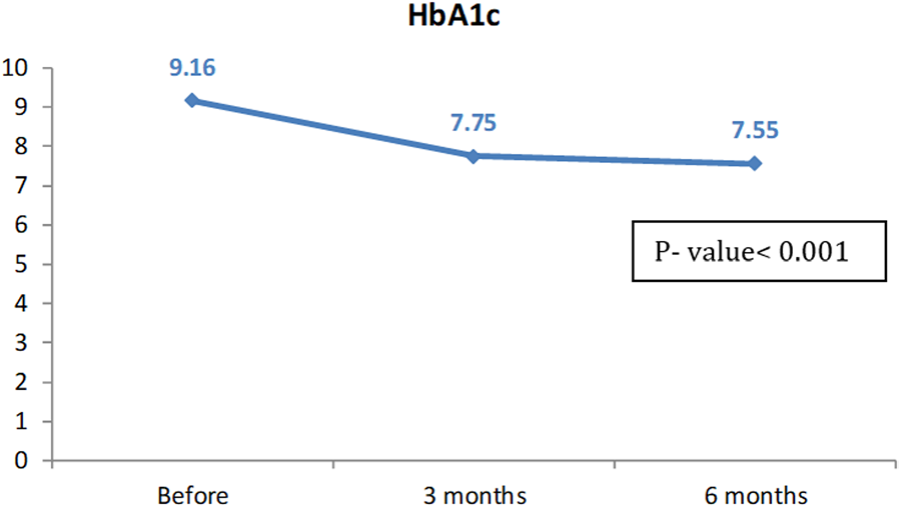
Comparison between HbA1c before pump, 3 months and 6 months later among the studied patients

**Fig. 3:**
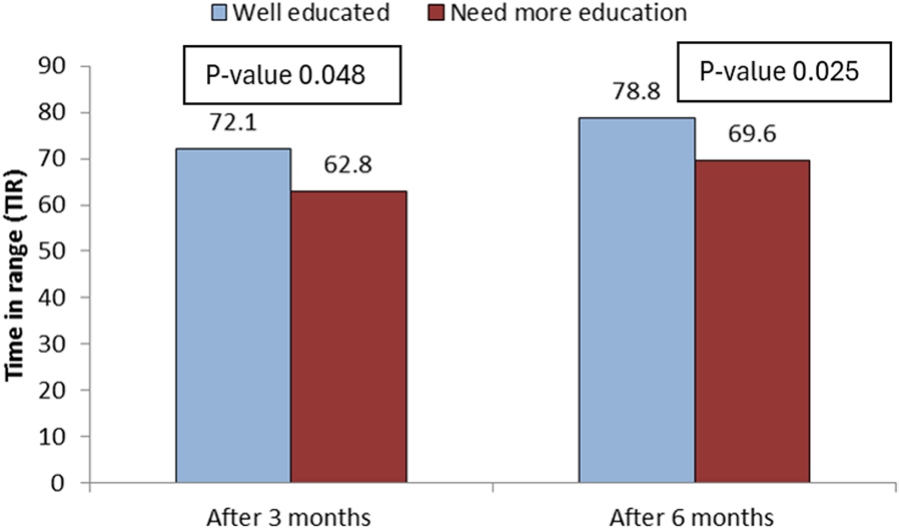
Comparison between studied patients regarding TIR 3 and 6 months after pump insertion

Regarding glycemic control outcomes, there was a statistically significant reduction in HbA1c over 6 months among participants who demonstrated good educational retention and adherence with p-value < 0.001 as illustrated in Fig. 2. Additionally, TIR improved significantly among well-educated children and CGM users compared to those who need more education with p-values 0.048 and 0.025 at 3 and 6 months respectively as illustrated in Fig. 3.

Comparing the two types of pumps used (715 and 780G) regarding glycemic control (HbA1c), it didn’t show statistically significant difference at baseline, 3 and 6 months as shown in Table 4.

**Table 4:** Comparison between pump types regarding HbA1c at different times among the studied patients

		Pump type		t-Test value	P-value	Sig.
		715	780 G			
		No. = 25	No. = 5			
HbA1C before pump	Mean ± SD	9.12 ± 1.67	9.36 ± 1.17	−0.299•	0.767	NS
	Median (IQR)	9 (8–9.8)	9.4 (8.7–9.8)			
	Range	6.2–12.9	7.9–11			
HbA1C 3 months after pump insertion	Mean ± SD	7.69 ± 0.66	8.06 ± 0.74	−1.124•	0.271	NS
	Median (IQR)	7.5 (7.2–8.2)	8.4 (8–8.5)			
	Range	6.9–9.4	6.8–8.6			
HbA1C 6 months after pump insertion	Mean ± SD	7.56 ± 0.87	7.52 ± 0.81	0.085•	0.933	NS
	Median (IQR)	7.4 (7–7.9)	7.3 (7–7.5)			
	Range	5.9–9.7	6.9–8.9			

P-value > 0.05: Non significant; P-value < 0.05: Significant; P-value < 0.01: Highly significant

•: Independent t-test

## Discussion

This study demonstrated that tailored diabetes education significantly enhances both adherence and glycemic control in children and adolescents with T1D initiating insulin pump therapy. The reduction in HbA1c and improvement in TIR observed in this cohort strongly support the integration of individualized training into clinical practice, especially when introducing advanced diabetes technologies such as CSII and CGM.

Educational intervention not only improved glycemic indices but also empowered patients and caregivers to manage insulin dosing, recognize and troubleshoot pump issues, and prevent complications. The improvement in diabetes knowledge scores at 3 and 6 months emphasizes the importance of ongoing reinforcement of educational content, particularly in pediatric populations where maturity and learning styles vary significantly.

Our findings are consistent with those of previous studies, highlighting the central role of education in optimizing outcomes with diabetes technology. For example, the ISPAD Clinical Practice Guidelines recommend individualized education as a cornerstone of pediatric T1D care. Moreover, real-world data from other regions have similarly shown that well-educated pump users achieve superior glycemic outcomes compared to those lacking structured instruction.

In agreement with the study conducted by *Ozgen et al.* [4], a total of 35 subjects participated in their prospective study. All patients were given insulin pump user retraining. Their knowledge level and application skills, metabolic parameters, quality of life, and satisfaction from treatment were evaluated at baseline and after 6 months. There was significant improvement in patients’ knowledge and application skills after insulin pump user retraining. HbA1c levels of patients with poor glycemic control improved after retraining (8.61% ± 0.78 vs. 8.23% ± 0.79, *p* = 0.02) [4].

The Current study agrees with another study conducted by *Beato-Víbora et al.* [5], who studied the effect of Implementation of an Advanced Hybrid Closed-Loop System in Adolescents and Adults with T1D on TIR. The study concluded that higher levels of diabetes education are associated with better glycemic control and improved overall management of the disease [5].

In the present study there was statistically significant improvement in ICR with P-value < 0.001 and ISF with P-value < 0.001 after shifting to insulin pump therapy among the studied patients. This was inconsistent with *Karges et al.* [6], which is a population-based cohort study comparing patients with T1D who used pump therapy and patients who used insulin injection therapy conducted in Germany; the study highlighted that total daily insulin dose was lower and prandial to total insulin ratio was higher in pump therapy compared with injection therapy, significant for all age groups of the matched cohort *P* < 0.001 [6].

The current study also highlighted some practical challenges, including device-related skin reactions and occasional technical errors. However, these were manageable and decreased over time with increased user experience and support.

In the present study there was a highly significant improvement of HbA1c after 6 months in patients on CSII either using flash glucose monitoring or SMBG especially among those who were well educated.

Improvements in CGM in recent years have changed the treatment of T1D by permitting the automation of glucose control [7].

In the current study, there was no significant difference between MiniMed 715 and MiniMed 780G pumps regarding hypoglycemia, hyperglycemia, cannula tenting, difficulties with carbohydrate counting, and set changes.

Moreover, there was significant improvement in TIR with P-value 0.048, 0.025 after 3 and 6 months respectively in well-educated patients. Agreeing with Alonso Martín et al. [8], who stated that an indication of good metabolic control in patients participating in the study was the absence of acute decompensation episodes with ketosis or severe hypoglycemia, as the result of adequate diabetes education, also improvement of patient’s technical skills after re-training program [8].

In Pacheco et al. [9] study, about 47 adults with T1D used a structured education program (SEP). The results showed that there was a significant reduction in glycated hemoglobin (HbA1c) levels by approximately 20% at one year and 11% at eight months after the program. Also, there was more than 70% improvement in TIR. This improvement in glycemic control was accompanied by increased knowledge about diabetes care and self-confidence in managing the disease [9].

Alassaf et al. [10]. conducted a retrospective study on a dietary structured education program for newly diagnosed children with T1D. They found that the program was associated with better glycemic control at six and twelve months after diagnosis. The program’s effectiveness was particularly evident in children older than five years and those with higher maternal educational levels [10].

Essien et al. [11].in a randomized control study, showed encouraging results of their structured education program intervention, on the glycemic control in Nigeria as an example of low-middle income countries [11].

## Conclusions

Tailored diabetes education is a fundamental component in the successful initiation and maintenance of insulin pump therapy in children and adolescents with T1D. This study highlights the critical role of tailored diabetes education in optimizing the outcomes of pediatric patients using CSII for T1D. The integration of individualized education significantly improved glycemic control, increased adherence, and enhanced disease-related knowledge among children and adolescents over a 6-month period.

Our findings support the premise that technology alone is insufficient to achieve optimal diabetes management outcomes. Instead, education tailored to the developmental and cognitive needs of pediatric patients and reinforced through structured follow-up remains indispensable for effective use of CSII and related diabetes technologies.

Implementing structured education programs as a routine part of pump therapy initiation and follow-up is essential in pediatric diabetes care. Wider application of such programs in diverse clinical settings could bridge knowledge gaps, reduce complications, and empower young patients to take ownership of their condition.

## Limitations

One limitation was the relatively small sample size and single-center design, which may affect generalizability. Additionally, the limited use of advanced closed-loop systems like the MiniMed 780G precluded more detailed subgroup analysis.

A primary limitation of our study is that the education program was delivered concurrently with the transition to CSII. Therefore, we cannot disentangle the individual effects of the new technology versus the educational intervention, although our findings demonstrate the powerful synergistic effect of combining both.

A notable observation is that meaningful improvements in glycemic control were achieved predominantly among patients utilizing older, non-automated technologies, underscoring the fundamental impact of structured diabetes education independent of advanced device integration.

Nevertheless, this study contributes valuable local evidence supporting structured, repeated diabetes education as a critical adjunct to CSII therapy in pediatric populations.

## Data Availability

All data generated and/or analysed during this study are available from the corresponding author on reasonable request.

## Abbreviations

T1D: Type 1 Diabetes
CSII: Continuous Subcutaneous Insulin Infusion
MDI: Multiple Daily Injection
ISPAD: International Society of Pediatric and Adolescent Diabetes
ADA: American Diabetes Association
CGM: Continuous Glucose Monitoring
TIR: Time In Range
ICR: Insulin Carbohydrate Ratio
ISF: Insulin Sensitivity Factor
SMBG: Self-Monitoring of Blood Glucose
RBS: Random blood sugar
SPSS: Statistical Package for the Social Sciences
